# Supplementary material for: Opening a Novel Biosynthetic Pathway to Dihydroxyacetone and Glycerol in Escherichia coli Mutants through Expression of a Gene Variant (fsaAA129S) for Fructose 6-Phosphate Aldolase
Source: Int J Mol Sci. 2020 Dec 17;21(24):9625. doi: 10.3390/ijms21249625 (PMC7767278; doi:10.3390/ijms21249625)
Supplement: Supplementary file 1 [file ijms-21-09625-s001.pdf]

Supplementary Material

Emma Guitart Font, Georg A. Sprenger

Supplementary Figure 1. SDS-PAGE of cfe and heat-treated cfe (HT) samples of LJ110 and GL3/pJF119*fsaA*

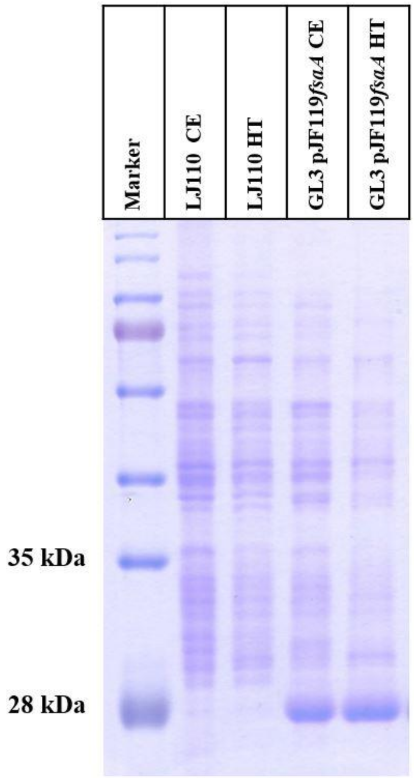

Samples of strains LJ110 and GL3/pJF119*fsaA* after overnight growth on MM with 28 mM fructose and 100 µM IPTG were taken. The band at 28 kDa corresponds to FSA wt.

Supplementary Figure 2. Progress over time of the $OD_{600\ nm}$ (**×**) and the concentrations of glucose (●), DHA (□) and glycerol (▲) when GL6 and GL7 were grown in shake flasks on MM with glucose and 100 μM IPTG.

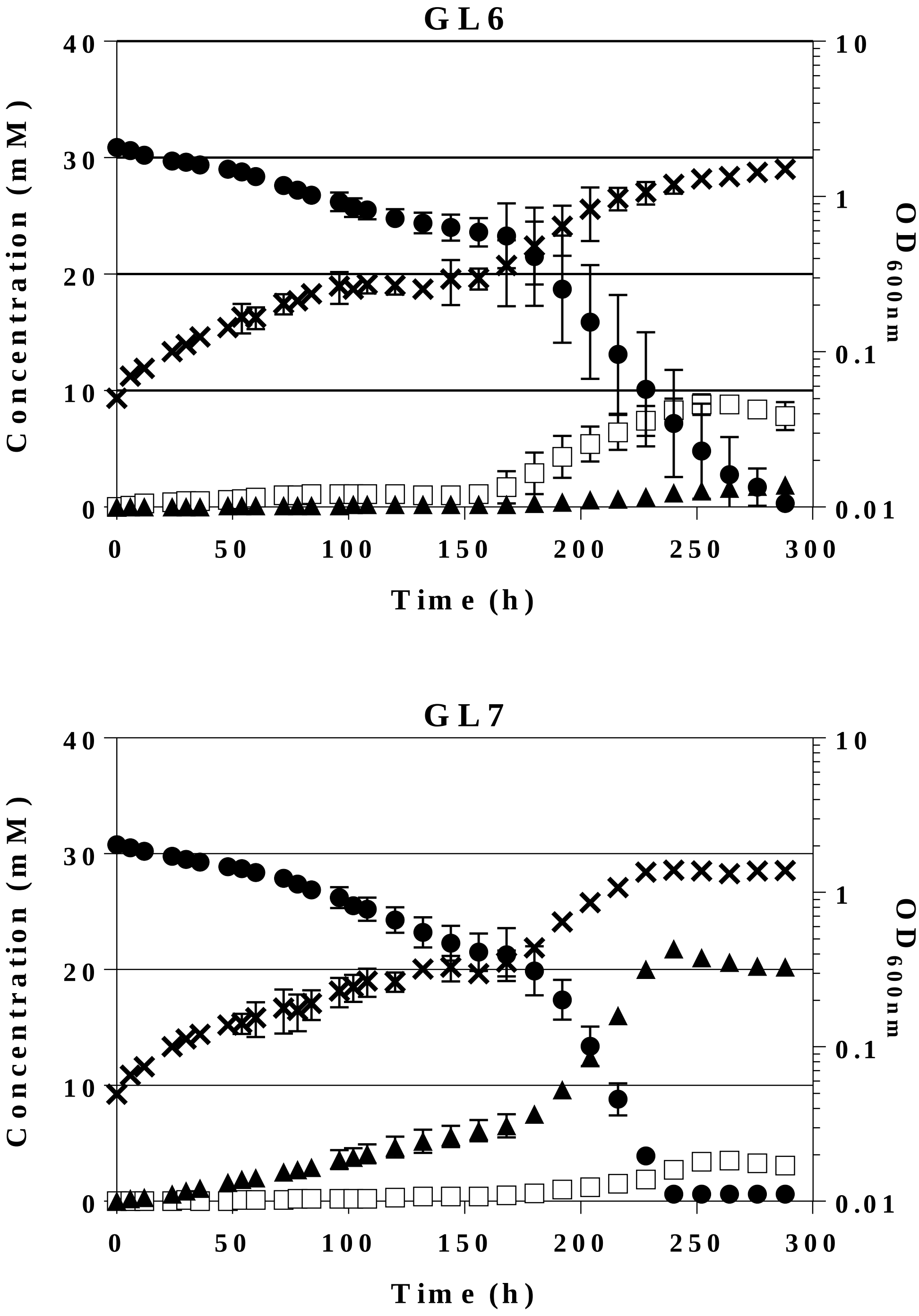

Supplementary Figure 3

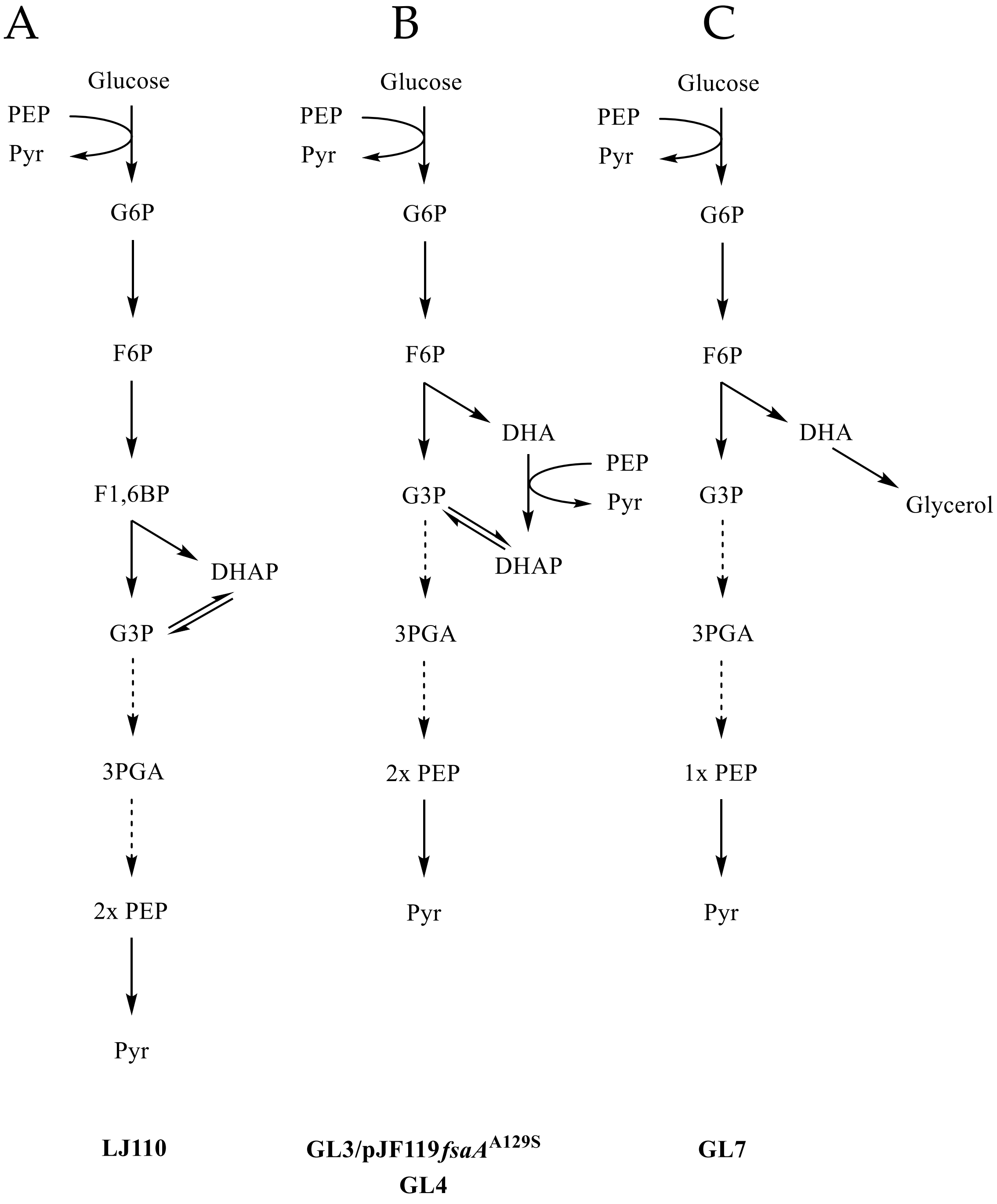

Different PEP consumption and formation routes in strains LJ110 (wild type, A), GL4 or GL3/-pJF*fsaA*^{A129S} (B), and GL7 (C). For a discussion see main text.

Supplementary Figure 4. Verification of the presence or deletion of the *zwf*, *pfkB*, *pfkA*, *dhaKLM*, *glpK,* or gene insertion/disruption of *rbsK* (for integration of P_{tac}-*gldA)* and *lacZ* (for integration of P_{tac}-*fsaA*^{A129S}*)* genes in the chromosome of LJ110 and of the GL-strains by colony PCR. LJ110 was used as control.

Δ*zwf*

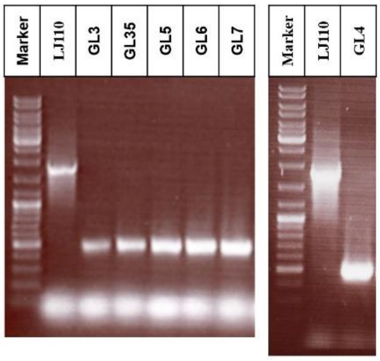

Δ*pfkB*

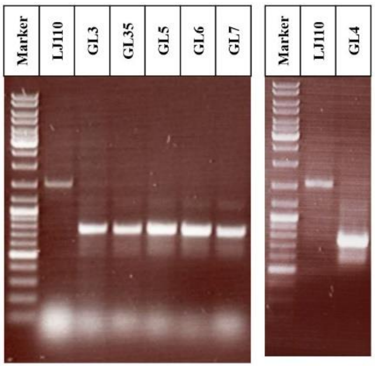

Δ*pfkA*

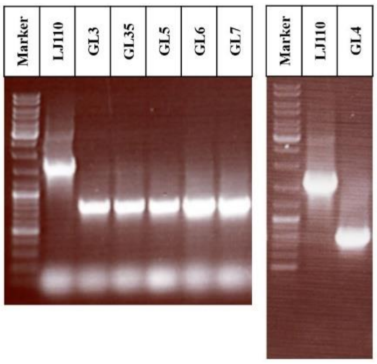

Δ*lacZ*::P_{tac}-*fsaA*^{A129S}

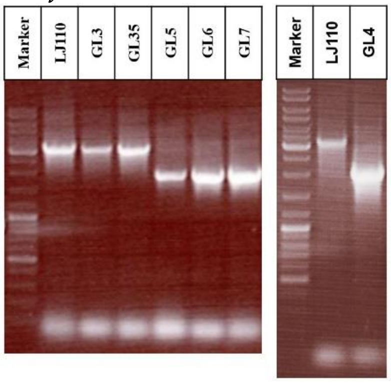

Δ*dhaKLM*

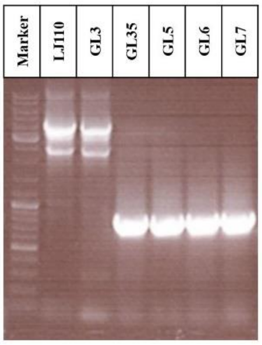

Δ*glpK*

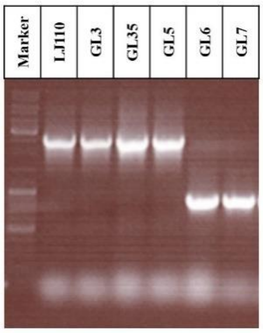

Δ*rbsK*::P_{tac}-*gldA*

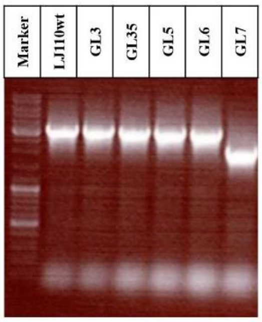

DNA size Marker

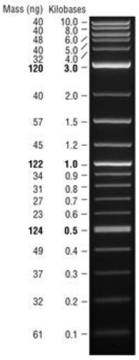

Supplementary Table 1

Oligonucleotides used for gene deletions (recombineering technique, [36]). The sequences homologous to pCO1-cat are underlined.

Name	Sequence (5′→ 3′)
zwf-del5′	GTTAGTTAACTTAAGGAGAATGACATGGCGGTAACGCAAATTGTGTAGGCTGGAGCTGCTTCG
zwf-del3′	ATTACTCAAACTCATTCCAGGAACGACCATCACGGGTAATCATCATATGAATATCCTCCTTAGTTC
pfkB-del5′	TATACGTTGACACTTGCGCCCTCTCTCGATAGCGCAACAATTATTGTGTAGGCTGGAGCTGCTTCG
pfkB-del3′	AATGCTGGGGGAATGTTTTTGTTAGCGGGAAAGGTAAGCGTAAACATATGAATATCCTCCTTAGTTC
pfkA-del5′	CAGATTTCATTTTGCATTCCAAAGTTCAGAGGTAGTCATGATTTGTGTAGGCTGGAGCTGCTTCG
pfkA-del3′	TTCCGAAATCATTAATACAGTTTTTCGCGCAGTCCAGCCAGCATATGAATATCCTCCTTAGTTC

Oligonucleotides used for gene deletions (CRISPR/Cas, [37). The inserted restriction sites are underlined (A_r oligonucleotides contained the restriction site for Bgl*II* and C_f for BamH*I*).

**Name**	**Sequence (5′→ 3′)**
A_f-dhaKLM-5′	ATATTCCCAGGCATCTTCCAGCGCAG
A_r-dhaKLM-3′	TTTTAGATCTAGCAATTACGGTAGGGCATGGATG
C_f-dhaKLM-5′	TTTTGGATCCTTCGGATGGCATCGTTCTGATGTC
C_r-dhaKLM-3′	AATAAAATATCAGGCGGCTGTGGTGTTAC
A_f-glpK-5′	AAAGTGTTGATCTGGCTGGCACTTTC
A_r-glpK-3′	TTTTAGATCTAGCTTTTTGTTCTGAAGGAGTTGTG
C_f-glpK-5′	TTTTGGATCCTACTGCTTAGAGTTTGCTATGAGAC
C_r-glpK-3′	TTCAGATCAATGGTGCCTTTGGCTC

Oligonucleotides used for gene integrations. The inserted restriction sites are underlined. P_{tac}-*fsaA*^{A129S} and P_{tac}-*gldA* were amplified from pJF119*fsaA*^{A129S} and pJF119ΔEP_{tac}*gldA*, respectively, by using the I_f-Ptac-gene (BstB*I*) and the I_r-Ptac-gene (Pst*I*) oligonucleotides. These fragments could be combined with either the A and C fragments of *lacZ* or the ones of *rbsK*.

**Name**	**Sequence (5′→ 3′)**
A_f-lacZ-5′ (EcoR*I*)	TTTTGAATTCTTCACACAGGAAACAGCTATGACC
A_r-lacZ-3′ (BstB*I*)	TTTTTTCGAAAGCGAGTAACAACCCGTCGGATTC
C_f-lacZ-5′ (Pst*I*)	TTTTCTGCAGATGGCGATTACCGTTGATGTTGAAG
C_r-lacZ-3′ (Hind*III*)	TTTTAAGCTTGCTCCAGGAGTCGTCGCCACCAATC
A_f-rbsK-5′ (EcoR*I*)	TTTTGAATTCAATGCAGAACCTGTTGACCGCTCATC
A_r-rbsK-3′ (BstB*I*)	TTTTTTCGAATAAAATGCGCCACCGTGTTAGGGTG
C_f-rbsK-5′ (Pst*I*)	TTTTCTGCAGTCAATAAAGATCGCTTCGTCAGTG
C_r-rbsK-3′ (Hind*III*)	TTTTAAGCTTAATAAGATCGCTGTCGCCATCGAAC
I_f-Ptac-gene-5′ (BstB*I*)	TTTTTTCGAATGGTATGGCTGTGCAGGTCGTAAATC
I_r-Ptac-gene-3′ (Pst*I*)	TTTTCTGCAGTTTTATCAGACCGCTTCTGCGTTC

Oligonucleotides for the construction of pTarget. For the amplification of each pTarget with a concret sgRNA, the "Universal pTarget" oligonucleotide was used in combination with the oligonucleotide containing the sgRNA in question (CRISPR). The sgRNA sequence is underlined.

**Name**	**Sequence (5'→ 3')**
Universal pTarget	ACTAGTATTATACCTAGGACTGAGCTAGC
dhaKLM (CRISPR)	TTGTGGATCGTCAATTCCCGGTTTTAGAGCTAGAAATAGCAAGTTAAAATAAGGCTAG
glpK (CRISPR)	GCACAACTGACCAAACAGCGGTTTTAGAGCTAGAAATAGCAAGTTAAAATAAGGCTAG
lacZ (CRISPR)	TCACCGCCGTAAGCCGACCACGTTTTAGAGCTAGAAATAGCAAGTTAAAATAAGGCTAG
rbsK (CRISPR)	GCTGCCATCACACTTTCGAGGTTTTAGAGCTAGAAATAGCAAGTTAAAATAAGGCTAG

Oligonucleotides used for screening of gene deletions and gene integrations in the chromosome with the expected amplicon length.

Name	Sequence (5′→ 3′)	Amplicon length	
		Wild-type	Mutant
Test-zwf-5′	CGCGCTTTTCCCGTAATCGCACG	1.8 kb	0.5 kb
Test-zwf-3′	TGAGTTGTCAGAGCAGGATGATTCAC		
Test-pfkB-5′	AAGGATCAAAGATTAGCGTCCCTGGAAAG	1.6 kb	0.8 kb
Test-pfkB-3′	TCTGTTGCTATTCCATTCCTCCCAGGTC		
Test-pfkA-5′	GAAGCTGAATATCCTTTGCCATAAC	1.6 kb	0.8 kb
Test-pfkA-3′	TATTTTACGGCGTTTTCCGGGATCG		
A_f-dhaKLM-5′	ATATTCCCAGGCATCTTCCAGCGCAG	3.6 kb	0.8 kb
C_r-dhaKLM-3′	AATAAAATATCAGGCGGCTGTGGTGTTAC		
Test-lacZ-5′	AAAAACCACCCTGGCGCCCAATAC	3.3 kb	2 kb (P_{tac}-*fsaA*^{A129S})
Test-lacZ-3′	AGACCAACTGGTAATGGTAGCGAC		
A_f-glpK-5′	AAAGTGTTGATCTGGCTGGCACTTTC	2.5 kb	0.8 kb
C_r-glpK-3′	TTCAGATCAATGGTGCCTTTGGCTC		
A_f-rbsK-5′	TTTTGAATTCAATGCAGAACCTGTTGACCGCTCATC	3 kb	2.5 kb
C_r-rbsK-3′	TTTTAAGCTTAATAAGATCGCTGTCGCCATCGAAC		

Oligonucleotides used for qPCR with the amplicon length and the location of the amplicon in the gene (beginning, middle or end).

Name	Sequence (5′→ 3′)	Amplicon length	Location
dhaK-5′-cDNA	AGACTGGAGCGGTTGTTTGG	207 bp	Middle
dhaK-3′-cDNA	CCTGGCGGATAATGAGATGG		
fsaA-5′-cDNA	GTAGCGGCATTCAGACTGTG	215 bp	End
fsaA-3′-cDNA	GCCAGTCCTGCTCAAACTTC		
fsaB-5′-cDNA	CGTTGCTCCGTATGTTAACC	197 bp	End
fsaB-3′-cDNA	TGTTGCGCTACATCTAAGGG		
ftsZ-5′-cDNA	TGCATTTGCTTCCGACAACG	111 bp	End
ftsZ-3′-cDNA	ACGTTTGTCCATGCCGATAC		
gapA-5′-cDNA	GACTATCAAAGTAGGTATCAACGGTTTT	148 bp	Beginning
gapA-3′-cDNA	GAGTGGAGTCATATTTCAGCATGTAT		
gldA-5′-cDNA	AAATTGCGCCGTTTGGCGGTG	450 bp	Middle
gldA-3′-cDNA	AGGGTGTTGTAGCACAGTTC		
glpK-5′-cDNA	GGCCGTGTCCATGTGACCGATTAC	194 bp	Middle
glpK-3′-cDNA	GGAGATTGGAATACGCGTGCCGCC		
lacI-5′-cDNA	ACGGCGGGATATAACATGAG	338 bp	Middle
lacI-3′-cDNA	ATCTGGTCGCATTGGGTCAC		
lacZ-5′-cDNA	CGAGTGGCAACATGGAAATC	295 bp	End
lacZ-3′-cDNA	TGAAAGCTGGCTACAGGAAG		

(Continuation)

Name	Sequence (5′→ 3′)	Amplicon length	Location
pfkA-5′-cDNA	TACGCTGGTGAGAAGAAGAG	263 bp	Middle
pfkA-3′-cDNA	AATTCCGCGACGAGAACATC		
pfkB-5′-cDNA	AGTTCTGGCGAAGCGTTAAG	303 bp	End
pfkB-3′-cDNA	CTGCCAGTTTCAGTGTCATC		
pgi-5′-cDNA	GGCGAAATTACAGGATCTGG	278 bp	Beginning
pgi-3′-cDNA	CCGATCCCGATGTTCACTAC		
ptsG-5′-cDNA	TTTGTGCCGATCATTTCTGGCCTG	241 bp	Middle
ptsG-3′-cDNA	ACCTGACCTGCTGCGTTGGTGTATTC		
sgrS-5′-cDNA	TGCCCCATGCGTCAGTTTTATCAGCAC	191 bp	Middle
sgrS-3′-cDNA	ATCTGCTGGCGGGTGATTTTACAC		
zwf-5′-cDNA	CAGCAGCAAACGTTCATAGG	256 bp	Beginning
zwf-3′-cDNA	GCCGACCAAATGTTCTGAAG		

Supplementary Table 2. Comparison of mRNA contents between wt LJ110 and GL4 during the exponential phase on MM with glucose and 100 µM IPTG. *ftsZ* was used as a calibrator. mRNA data from two independent biological replicates each with three technical replicates. *ftsZ* (cell division protein), *gapA* (glyceraldehyde 3-phosphae dehydrogenase A), *zwf* (glucose 6-phosphate dehydrogenase), *pfkA* (phosphofructokinase A), *pfkB* (phosphofructokinase B), *lacZ* (β-galactosidase), *dhaK* (dihydroxyacetone kinase), *fsaB* (fructose 6-phosphate aldolase B), *gldA* (glycerol dehydrogenase), *sgrS* (sugar transport-related sRNA), *ptsG* (glucose-specific PTS enzyme), *glpK* (glycerol kinase), *lacI* (lactose inhibitor), *fsaA* (fructose 6-phosphate aldolase A).

**Genes**		**LJ110**		**GL4**	
		**Average**	**St. deviation**	**Average**	**St. deviation**
***ftsZ* as calibrator**	***ftsZ***	1.0	0.0	1.0	0.0
	gapA	9.6	1.4	3.0	0.9
	zwf	0.4	0.1	0.0	0.0
	pfkA	1.4	1.3	0.0	0.0
	pfkB	0.0	0.0	0.0	0.0
	lacZ	7.0	2.8	0.0	0.0
	dhaK	0.8	0.7	2.9	1.4
	fsaB	0.0	0.0	0.0	0.0
	gldA	0.0	0.0	0.0	0.0
	sgrS	0.0	0.0	0.2	0.0
	ptsG	1.7	0.5	0.4	0.2
	glpK	0.1	0.0	0.1	0.0
	lacI	0.1	0.0	0.1	0.0
	fsaA	0.1	0.0	20.1	1.8
